# The Lung Cancer Immune Prognostic Score predicts pathologic complete response and survival in NSCLC patients receiving neoadjuvant immunochemotherapy

**DOI:** 10.3389/fimmu.2025.1567565

**Published:** 2025-04-16

**Authors:** Yuyan Xie, Zhihao Shi, Tong Chen, Hongyan Li, Menglin Fan, Xuqin Xiang, Fang Liu

**Affiliations:** Department of Medical Oncology, Harbin Medical University Cancer Hospital, Harbin, China

**Keywords:** neoadjuvant immunochemotherapy, non-small cell lung cancer, pathologic complete response, inflammatory and nutritional indices, prognostic factors

## Abstract

**Introduction:**

Neoadjuvant immunochemotherapy (nICT) has significantly improved event-free survival (EFS) and pathologic complete response (pCR) in patients with resectable non-small cell lung cancer (NSCLC). However, the lack of validated biomarkers limits their ability to predict therapeutic efficacy and survival outcomes. This study aimed to develop a novel inflammatory and nutritional index, the Lung Cancer Immune Prognostic Score (LCIPS), to predict pCR and survival prognosis in patients with NSCLC.

**Methods:**

This retrospective study included 131 patients with clinical stage IB-IIIB NSCLC who underwent neoadjuvant immunochemotherapy between May 2020 and May 2024. Baseline clinical data and hematological parameters were collected. Lasso regression analysis was employed to identify hematological indices associated with pCR, and the LCIPS was constructed based on these factors. Kaplan-Meier survival analysis and log-rank tests were used to assess survival differences. Logistic regression was performed to identify the predictors of pCR, while Cox regression analysis determined independent prognostic factors for disease-free survival (DFS) and overall survival (OS). The predictive performance of the LCIPS was validated using a nomogram.

**Results:**

Lasso regression identified three core hematological indices: the albumin-to-globulin ratio (A/G), absolute monocyte count (MONO), and absolute lymphocyte count (LYM). The LCIPS formula was as follows: LCIPS=0.900×A/G+0.761×MONO (10^9^/L) −0.065×LYM (10^9^/L). Receiver operating characteristic (ROC) curve analysis showed that the LCIPS had superior predictive efficacy (area under the curve (AUC) = 0.68) compared to other classical markers. Univariate and multivariate logistic regression analyses identified intraoperative lymph node dissection status and A/G and LCIPS as independent predictors of pCR (*p* < 0.05). Multivariate Cox regression analysis demonstrated that smoking status and LCIPS were independent prognostic factors for DFS and OS. Nomogram validation indicated robust predictive accuracy for LCIPS. Notably, among immune-related adverse events (irAEs), endocrine- and cardiac-related irAEs significantly affected DFS (*p* < 0.05).

**Discussion:**

LCIPS is an independent predictor of pCR in patients with NSCLC receiving neoadjuvant immunochemotherapy and is associated with improved DFS and survival outcomes. This novel index offers valuable guidance for personalized treatment strategies.

## Graphical Abstract

Created with BioRender (https://biorender.com/).
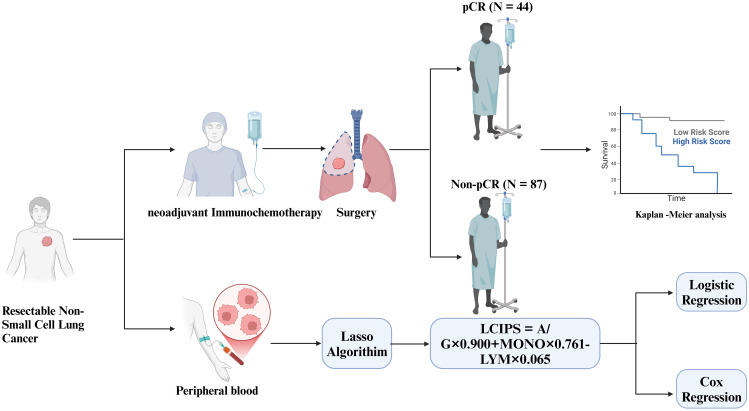

## 1 Introduction

Lung cancer is the leading cause of cancer-related morbidity and mortality worldwide and poses a critical public health challenge (1). Surgery remains the cornerstone of treatment for early-stage NSCLC, with a 1-year relapse-free survival rate of 96% in stage I patients after resection (2, 3). However, for patients with stage I-II NSCLC undergoing complete resection, the 5-year cumulative recurrence rate reaches 20%, with approximately 82% of recurrences occurring at distant sites, leading to poor survival outcomes (4). Improving long-term survival and reducing recurrence and metastasis remain pressing clinical challenges in patients with resectable NSCLC.

Neoadjuvant chemotherapy, which has historically been used to improve outcomes, has shown limited efficacy, with an absolute survival benefit of only approximately 5% (5). Recently, the advent of immunotherapy, particularly immune checkpoint inhibitors (ICIs), has revolutionized the treatment paradigm for resectable NSCLC (6). Notably, the CheckMate-816 trial demonstrated that nICT significantly improves EFS and pCR rates compared to chemotherapy alone without increasing the rate of adverse event rates (7). Moreover, a recent meta-analysis confirmed the superiority of nICT over chemotherapy alone in terms of surgical outcomes, pathological response, and treatment efficacy (8). However, the number of large-scale clinical trials evaluating nICT remains limited and further validation of its efficacy is warranted.

Despite these promising outcomes, predictive biomarkers for nICT efficacy are yet to be established. Although PD-L1 expression has been identified as a potential predictive marker, its role in early-stage NSCLC remains controversial (9). For example, trials such as NCT02259621 (10), CTONG1804 (11), and AEGEAN (12) have suggested a correlation between PD-L1 expression and pathological response. However, its clinical utility has not been definitively endorsed by guidelines, such as those from the National Comprehensive Cancer Network (NCCN) or Food and Drug Administration. Additionally, inflammatory markers (13, 14) and nutritional markers (15, 16) have shown potential for predicting nICT efficacy. However, single markers or simple parameter combinations often fail to fully capture the complexity of the therapeutic responses.

In this study, we retrospectively analyzed 131 patients with NSCLC undergoing nICT to identify predictors of pCR and developed a novel inflammatory and nutritional index, LCIPS, based on hematological parameters. Furthermore, we systematically evaluated the survival prognosis of these patients to provide new insights into the efficacy of nICT and to support stratified patient management for improved outcomes.

## 2 Materials and methods

### 2.1 Patient selection

We retrospectively collected clinical data from 131 patients with lung cancer who received nICT between May 2020 and May 2024 at the Affiliated Cancer Hospital of Harbin Medical University. Data were retrieved from electronic medical records in compliance with the relevant data protection and privacy regulations. All personal information was handled in accordance with the ethical guidelines of the 1964 Declaration of Helsinki and its subsequent amendments. This study was approved by the Institutional Review Board of the Harbin Medical University Cancer Hospital (Ethical approval number: AF-27-2.6).

The inclusion criteria were as follows: (1) patients pathologically diagnosed with lung cancer at clinical stages IB-IIIB; (2) patients receiving neoadjuvant immunochemotherapy prior to surgery; (3) resectable lesions confirmed by preoperative evaluation conducted by three or more senior thoracic surgeons; and (4) an ECOG performance status of 0–1, indicating tolerability to nICT.

Exclusion criteria included: (1) incomplete clinical data, particularly missing hematological results; (2) concurrent malignancies; (3) prior exposure to antitumor therapies, including chemotherapy, radiotherapy, or targeted treatments, within 4 weeks before initiating PD-1 inhibitors; (4) lack of validated efficacy assessments; and (5) systemic diseases precluding surgical intervention.

### 2.2 Pharmacological treatment

Patients received at least two cycles of nICT, administered once every 21 days. The immunotherapeutic agents used included five programmed cell death protein 1 (PD-1) inhibitors: Camrelizumab (200 mg), Sintilimab (200 mg), Tislelizumab (200 mg), Toripalimab (240 mg), and Pembrolizumab (200 mg). The choice of specific agents was based on clinical indications and the patient’s financial status.

### 2.3 Data collection and follow-up

We retrospectively collected clinical and medical records of all patients from the electronic medical record system. Hematological parameters and baseline clinical information were obtained within one week before the initiation of nICT.

The collected hematological parameters included lymphocyte count (LYM, 10^9^/L), neutrophil count (NEU, 10^9^/L), platelet count (PLT, 10^9^/L), monocyte count (MONO, 10^9^/L), eosinophil percentage (EOS%), total cholesterol (T-CHOL, mmol/L), albumin (ALB, g/L), albumin/globulin ratio (A/G), lactate dehydrogenase (LDH, U/L), glucose (Glu, mmol/L), squamous cell carcinoma antigen (SCC, ng/mL), carcinoembryonic antigen (CEA, ng/mL), neuron-specific enolase (NSE, ng/mL), carbohydrate antigen 19-9 (CA19-9, ng/mL), and several hematological indices, including the neutrophil-to-lymphocyte ratio (NLR = neutrophils/lymphocytes) (17), platelet-to-lymphocyte ratio (PLR = platelets/lymphocytes) (17, 18), lymphocyte-to-monocyte ratio (LMR = lymphocytes/monocytes) (17), prognostic nutritional index (PNI = albumin (g/dL) + 5 × lymphocytes (10^9^/L)) (19), and systemic immune-inflammation index (SII = platelets (10^9^/L) × neutrophils (10^9^/L)/lymphocytes (10^9^/L)) (20).

Baseline clinical information included sex, age, body mass index (BMI), primary tumor location, laterality, T stage, N stage, TNM stage, histological subtype, number of treatment cycles, lymph node dissection status, programmed death-ligand 1 (PD-L1) expression status, type of PD-1 inhibitor administered, and pathological response.

Additionally, all enrolled patients were followed up every three months through outpatient visits or telephone interviews, during which hematological and imaging examinations were conducted. Follow-up time was defined as the period from the date of surgery to either death from any cause or the last follow-up (November 1, 2024), whichever occurred first. The median follow-up duration for this study was 26.84 months (range: 5.7–55.0 months).

### 2.4 Study endpoints

Chest computed tomography (CT) was performed every two treatment cycles. Postoperative pathological evaluation of pCR was conducted by two specialized pathologists according to the multidisciplinary recommendations of the International Association for the Study of Lung Cancer (21). Pathologic complete response was defined as the complete absence or minimal presence of residual tumor cells (22). The primary study endpoint was pCR, whereas secondary endpoints included irAEs, DFS, and OS. DFS was defined as the interval from treatment initiation to recurrence or death from disease progression, while OS was defined as the interval from treatment initiation to death or the last follow-up.

### 2.5 Statistical analysis

Statistical analyses were conducted using the R software 4.2 (Vienna, Austria, https://cran.r-project.org), SPSS 27 (Chicago, IL, USA, https://www.ibm.com/spss), and GraphPad Prism 10(San Diego, CA, USA, https://www.graphpad.com/). Continuous variables following a Gaussian distribution were expressed as mean ± standard deviation (mean ± SD) and compared using independent sample t-tests. Non-normally distributed variables are presented as medians with interquartile ranges (median [IQR]) and compared using the Mann-Whitney U test. Categorical variables are expressed as counts and percentages (n, %), and differences were assessed using chi-square or Fisher’s exact tests. Survival outcomes (DFS and OS) were estimated using Kaplan-Meier curves, and differences were assessed using the log-rank test. Univariate and multivariate Cox proportional hazards regression models were used to identify independent prognostic factors with hazard ratios (HRs) and 95% confidence intervals (CIs). Predictors of pCR were evaluated using univariate and multivariate logistic regression analyses, incorporating key clinical variables such as age, sex, TNM stage, LCIPS, and smoking status. Lasso regression (Using the R package glmnet, version 4.1-8) (23) was applied to identify the optimal hematological parameters predicting nICT efficacy and prognosis. To avoid overfitting, the optimal cut-off value for LCIPS was determined through 10-fold cross-validation. The dataset was randomly split into 10 subsets, with 9 subsets used for model training and 1 subset for validation. This process was repeated 100 times, and the median cut-off value with the highest Youden index across iterations was selected as the final threshold. Model stability was assessed using the internal bootstrap method (n = 1000), and calibration curves (R package rms, version 6.8-2) (24) were constructed to evaluate model consistency. Diagnostic efficacy was assessed by calculating the AUC from the ROC curves(R package pROC, version1.18.5) (25). The nomogram’s predictive accuracy was validated using the C-index and clinical utility was evaluated using decision curve analysis (DCA) (R package dcurves, version0.5.0) (26). To assess the association between specific irAE types and survival outcomes, a correlation heatmap was generated using Spearman’s rank correlation analysis. Heatmap construction was performed with the pheatmap package (version 1.0.12) (27), incorporating normalized correlation coefficients (r) and their corresponding p-values. To address the issue of multiple hypothesis testing, the Benjamini-Hochberg false discovery rate (FDR) correction was applied for all analyses involving multiple comparisons. Adjusted *p*-values (q-values) <0.1 were considered statistically significant. Sensitivity analyses were performed to ensure robustness across the different models.

## 3 Results

### 3.1 Patient characteristics

**Figure 1:**
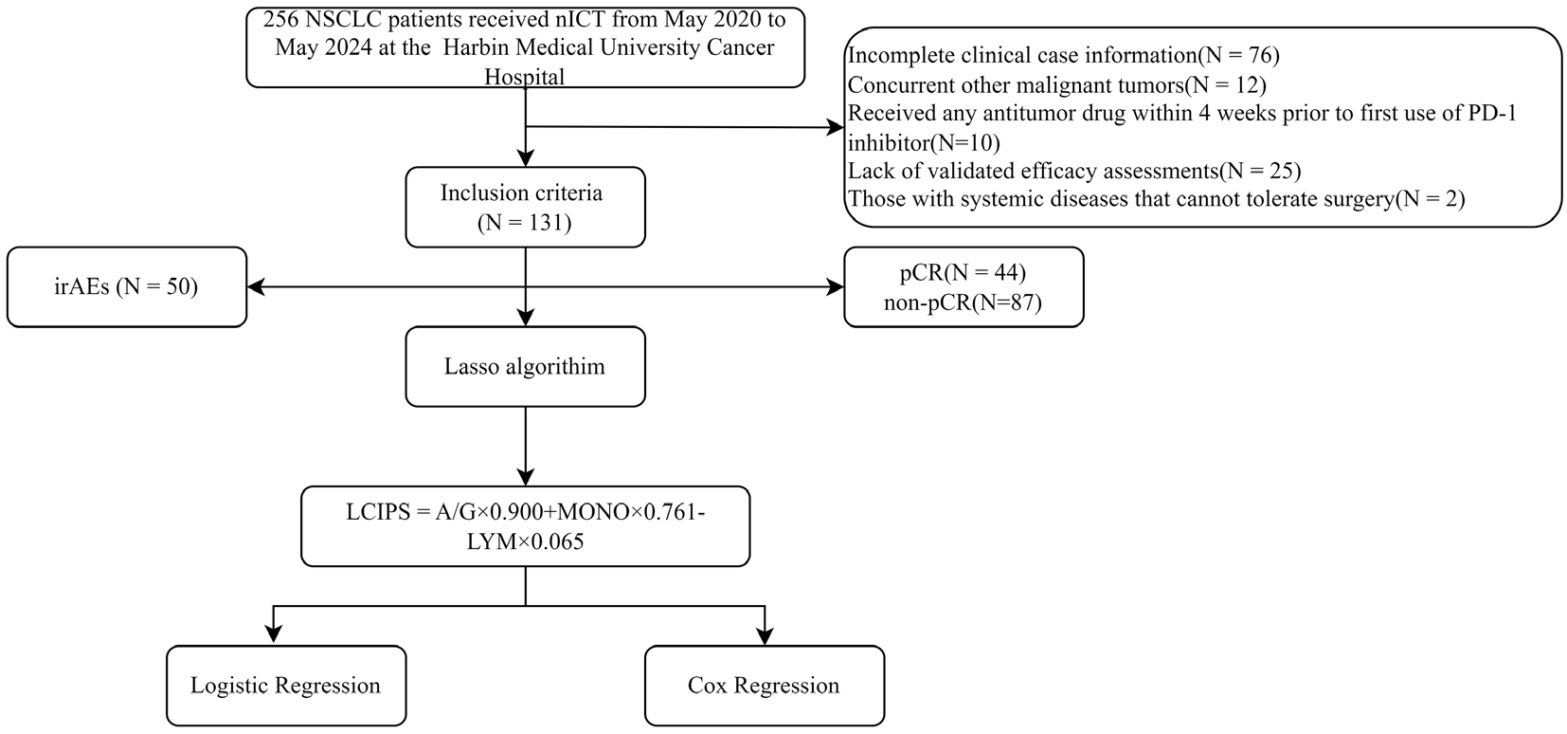
Flow chart of the study population.

A total of 131 patients with NSCLC were included in the study (**Figure 1**). Among them, 102 (77.86%) were male and 29 (22.14%) were female, with a median age of 62.5 years and a median body mass index (BMI) of 24.221(**Table 1**). The majority of lesions (n = 74) were located in the right lung, and 64 patients underwent two cycles of nICT. Most patients (n = 98) were diagnosed with squamous cell carcinoma, and stage III NSCLC (58.02%, n = 76) was the most common stage according to the TNM staging. Of the total cohort, 44 (33.59%) achieved pCR after nICT. Age was the only baseline characteristic that showed a significant difference (*p* = 0.019), while no other clinical or hematological parameters (**Table 2**) were statistically significant.

**Table 1:** Baseline clinical characteristics of patients.

Characteristics	Level	Total	non-pCR	pCR	p-Value
		(N=131)	(N=87)	(N=44)	
Sex (%)	Male	102 (77.86%)	73 (83.91%)	29 (65.91%)	0.019
	Female	29 (22.14%)	14 (16.09%)	15 (34.09%)	
Age (median [IQR])		62.50 (57.00-67.00)	63.00 (57.00-68.00)	62.00 (56.00-66.00)	0.582
Smoking (%)	Yes	62 (47.33%)	45 (51.72%)	17 (38.64%)	0.156
	No	69 (52.67%)	42 (48.28%)	27 (61.36%)	
BMI (%) (median [IQR])		24.221 (22.024-26.062)	23.951 (22.032-25.826)	24.643 (21.341-26.417)	0.183
Primary Site	Upper	61 (46.56%)	37 (42.53%)	24 (54.55%)	0.065
	Middle	9 (6.87%)	9 (10.34%)	0	
	Inferior	61 (46.56%)	41 (47.13%)	20 (45.45%)	
Laterality	Left	57 (43.51%)	35 (40.23%)	22 (50.00%)	0.287
	Right	74 (56.49%)	52 (59.77%)	22 (50.00%)	
T-Stage	1	11 (8.40%)	7 (8.05%)	4 (9.09%)	0.403
	2	62 (47.33%)	39 (44.83%)	23 (52.27%)	
	3	39 (29.77%)	30 (34.48%)	9 (20.45%)	
	4	19 (14.50%)	11 (12.64%)	8 (18.18%)	
N-Stage	0	37 (28.24%)	24 (27.59%)	13 (29.55%)	0.715
	1	37 (28.24%)	27 (31.03%)	10 (22.73%)	
	2	53 (40.46%)	34 (39.08%)	19 (43.18%)	
	3	4 (3.05%)	2 (2.30%)	2 (4.55%)	
TNM Stage	I	8 (6.11%)	5 (5.75%)	3 (6.82%)	0.938
	II	47 (35.88%)	32 (36.78%)	15 (34.09%)	
	III	76 (58.02%)	50 (57.47%)	26 (59.09%)	
Histological type	LUAD	30 (22.90%)	20 (22.99%)	10 (22.73%)	0.456
	LUSC	98 (74.81%)	64 (73.56%)	34 (77.27%)	
	Others	3 (2.29%)	3 (3.45%)	0	
Neoadjuvant cycles	2	64 (48.85%)	43 (49.43%)	21 (47.73%)	0.786
	3	43 (32.82%)	27 (31.03%)	16 (36.36%)	
	≥4	24 (18.32%)	17 (19.54%)	7 (15.91%)	
lymph node dissection	Yes	25 (19.08%)	15 (17.24%)	10 (22.73%)	0.45
	No	106 (80.92%)	72 (82.76%)	34 (77.27%)	
PD-L1 (%)	<1%	5 (3.82%)	4 (4.60%)	1 (2.27%)	0.348
	1-49%	9 (6.87%)	4 (4.60%)	5 (11.36%)	
	≥50%	6 (4.58%)	3 (3.45%)	3 (6.82%)	
	Unknown	111 (84.73%)	76 (87.36%)	35 (79.55%)	
PD-1 inhibitor	Camrelizumab	64 (48.85%)	45 (51.72%)	19 (43.18%)	0.416
	Sintilimab	30 (22.90%)	19 (21.84%)	11 (25.00%)	
	Tislelizumab	21 (16.03%)	15 (17.24%)	6 (13.64%)	
	Toripalimab	10 (7.63%)	4 (4.60%)	6 (13.64%)	
	Pembrolizumab	6 (4.58%)	4 (4.60%)	2 (4.55%)	

IQR, interquartile range; BMI, body mass index; LUAD, lung adenocarcinoma; LUSC, lung squamous carcinoma.

**Table 2:** Blood parameters.

Characteristics	Total	non-pCR	pCR	p-Value
	(N=131)	(N=87)	(N=44)	
LYM (10^9^/L, median (IQR))	1.910 (1.585-2.275)	1.870 (1.570-2.220)	1.940 (1.670-2.400)	0.324
NEU (10^9^/L, median (IQR))	4.550 (3.415-5.930)	4.545 (3.420-5.760)	4.690 (3.410-6.130)	0.766
PLT (10^9^/L, median (IQR))	265 (225-322)	260 (223-324)	267 (228-322)	0.981
MONO (10^9^/L, median (IQR))	0.520 (0.390-0.630)	0.515 (0.390-0.610)	0.540 (0.420-0.630)	0.321
EOS% (median (IQR))	1.800 (0.950-2.950)	1.850 (0.900-2.500)	1.700 (1.000-3.500)	0.921
T-CHOL (mmol/L,median (IQR))	4.880 (3.945-5.600)	4.900 (4.030-5.650)	4.580 (3.680-5.560)	0.780
ALB (g/L,median (IQR))	41.100 (37.950-43.100)	41.200 (37.900-43.000)	40.900 (38.000-43.300)	0.320
A/G (median (IQR))	1.300 (1.100-1.500)	1.300 (1.200-1.500)	1.200 (1.000-1.400)	0.053
LDH (U/L,median (IQR))	177 (156-204)	178 (160-202)	169 (152-207)	0.327
Glu (mmol/L,median (IQR))	5.200 (4.800-5.800)	5.100 (4.600-5.700)	5.400 (5.000-6.000)	0.044
SCC (ng/mL,median (IQR))	1.400 (0.900-2.500)	1.500 (0.900-2.500)	1.300 (0.900-2.500)	0.769
CEA (ng/mL,median (IQR))	3.305 (1.970-5.620)	3.300 (1.970-6.030)	3.380 (2.020-5.520)	0.847
NSE (ng/mL,median (IQR))	15.950 (13.510-17.750)	16.350 (14.300-18.320)	15.230 (12.580-17.570)	0.052
CA199 (ng/mL,median (IQR))	16.425 (10.170-23.320)	16.580 (10.170-29.300)	15.560 (10.550-18.900)	0.685
NLR	2.390 (1.706-3.207)	2.389 (1.695-3.204)	2.437 (1.774-3.210)	0.780
PLR	139.770 (111.065-186.065)	140.020 (112.570-190.910)	138.490 (105.160-175.120)	0.545
LMR	3.840 (2.840-4.970)	3.920 (3.000-5.140)	3.760 (2.820-4.690)	0.567
PNI	50.950 (47.000-53.850)	50.325 (46.300-53.800)	52.250 (48.450-54.000)	0.140
SII	616.150 (408.635-979.435)	620.430 (369.340-1066.660)	583.220 (451.810-847.010)	0.919

LYM, lymphocytes; NEU, neutrophils; PLT, platelets; MONO, monocytes; EOS%, eosinophil%; T-CHOL, total cholesterol; ALB, albumin; A/G, albumin globulin ratio; LDH, lactate dehydrogenase; Glu, glucose; SCC, squamous cell carcinoma antigen; CEA, carcinoembryonic antigen; NSE, neuron-specific enolase; CA199, carbohydrate antigen 199; NLR, neutrophil–lymphocyte ratio; PLR, platelet–lymphocyte ratio; LMR, lymphocyte-monocyte ratio; PNI, prognostic nutritional index; SII, systemic immune inflammation index.

### 3.2 Construction of LCIPS

**Figure 2:**
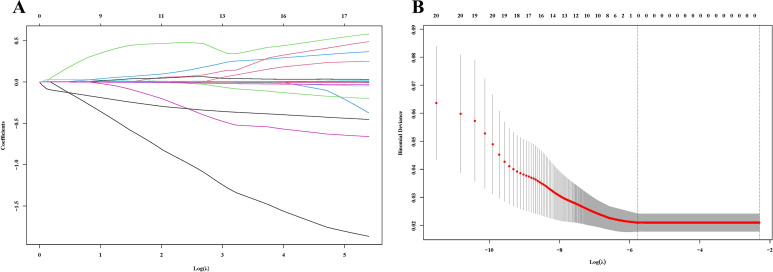
Lasso regression analysis. **(A)** The variation characteristics of the coefficient of variables; **(B)** The selection process of the optimum value of λ.

Lasso regression analysis was performed to identify hematological parameters associated with pathological response and survival prognosis after nICT. After 418 cross-validation cycles with an optimal penalty parameter λ of 0.0013, three core hematological indices were selected: A/G, MONO, and LYM (**Figure 2**).

The selection of these parameters was not only statistically robust but also biologically plausible, as each component reflects distinct aspects of immune regulation and tumor-host interactions: A/G ratio is a widely used indicator for assessing nutritional status and inflammatory responses. The levels of serum albumin and globulin influence inflammation and immune function, with hypoalbuminemia and hyperglobulinemia commonly considered markers of chronic inflammation in cancer patients. Albumin reflects the nutritional status of the body, while globulin is associated with immune and inflammatory responses (28, 36). Monocytes and their derived macrophages play a crucial role in immune responses. Studies have shown that cancer significantly disrupts the distribution of monocyte subsets and their transcriptome, which correlates with patient prognosis (29). In mouse cancer models, monocytes have been shown to contribute to tumor progression, metastasis, and resistance to anti-vascular endothelial growth factor therapy (30, 31). Lymphopenia has been significantly associated with immune treatment responses. Caux et al. found that lymphopenia correlates with both survival rates and chemotherapy toxicity in patients. Furthermore, lymphopenia also holds prognostic value for progression-free survival and OS (32).

The Lasso regression model directly identifies the most predictive variables through regularization, rather than using forward selection, backward selection, or stepwise regression methods. The β-coefficients for these indices were 0.900, 0.761, and -0.065, respectively. A novel inflammatory and nutritional index, LCIPS, was developed based on these parameters, with the formula: LCIPS=0.900×A/G+0.761×MONO (10^9^/L) −0.065×LYM (10^9^/L).

### 3.3 Comparison of LCIPS with other indicators

**Figure 3:**
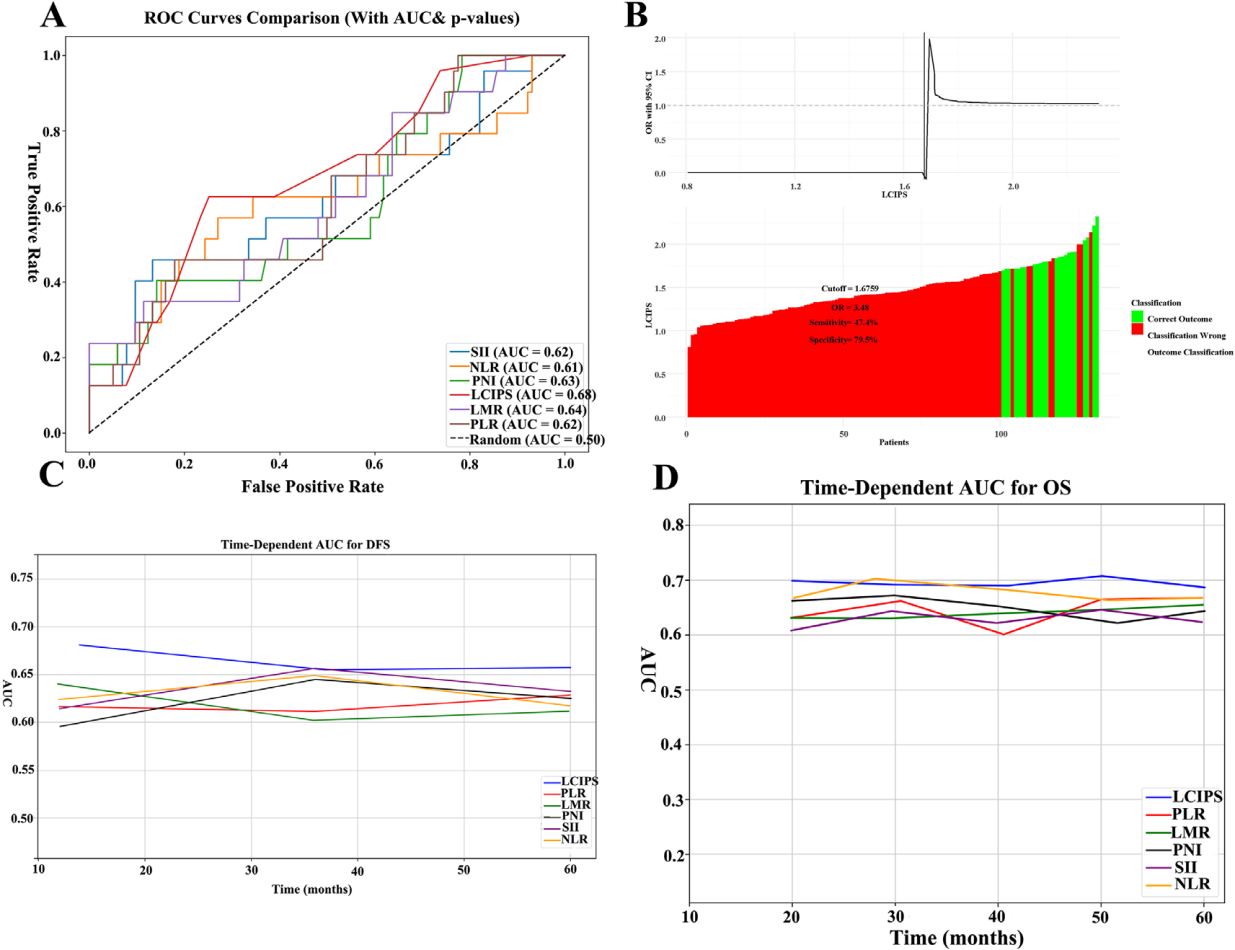
ROC curves of all markers. **(A)** The ROC comparisons between LCIPS and other indicators; **(B)** The optimum cut-off value based on the cutoff finder for LCIPS; **(C)** The comparison of time–ROC curves with different indicators for DFS.; **(D)** The comparison of time–ROC curves with different indicators for OS.

The predictive performance of the LCIPS was compared to that of other classical inflammatory and nutritional markers. ROC curve analysis demonstrated that the LCIPS achieved the highest discriminative ability among all evaluated indices (AUC = 0.68, 95% CI:0.652-0.752) (**Figure 3**; **Supplementary Table 1**). The optimal cut-off value for the LCIPS was determined through rigorous 10-fold cross-validation (100 repeats) to mitigate overfitting, yielding a threshold of 1.676 (sensitivity = 0.474, specificity = 0.795), which stratified patients into high-LCIPS (n = 32) and low-LCIPS (n = 99) groups.

Bootstrap resampling (n = 1,000) demonstrated moderate internal consistency (mean AUC = 0.657, 95% CI: 0.562–0.753), indicating that the LCIPS maintains reasonable discriminative capacity within the original cohort. Meanwhile, 10-fold cross-validation (100 repeats) revealed moderate generalizability (mean AUC = 0.728, 95% CI: 0.572–0.924). While the wide confidence interval in cross-validation may reflect limited sample size or outcome heterogeneity, the consistent median cut-off (1.676) across iterations suggests robust threshold stability.

### 3.4 Predictors of pCR using logistic regression analysis

**Figure 4:**
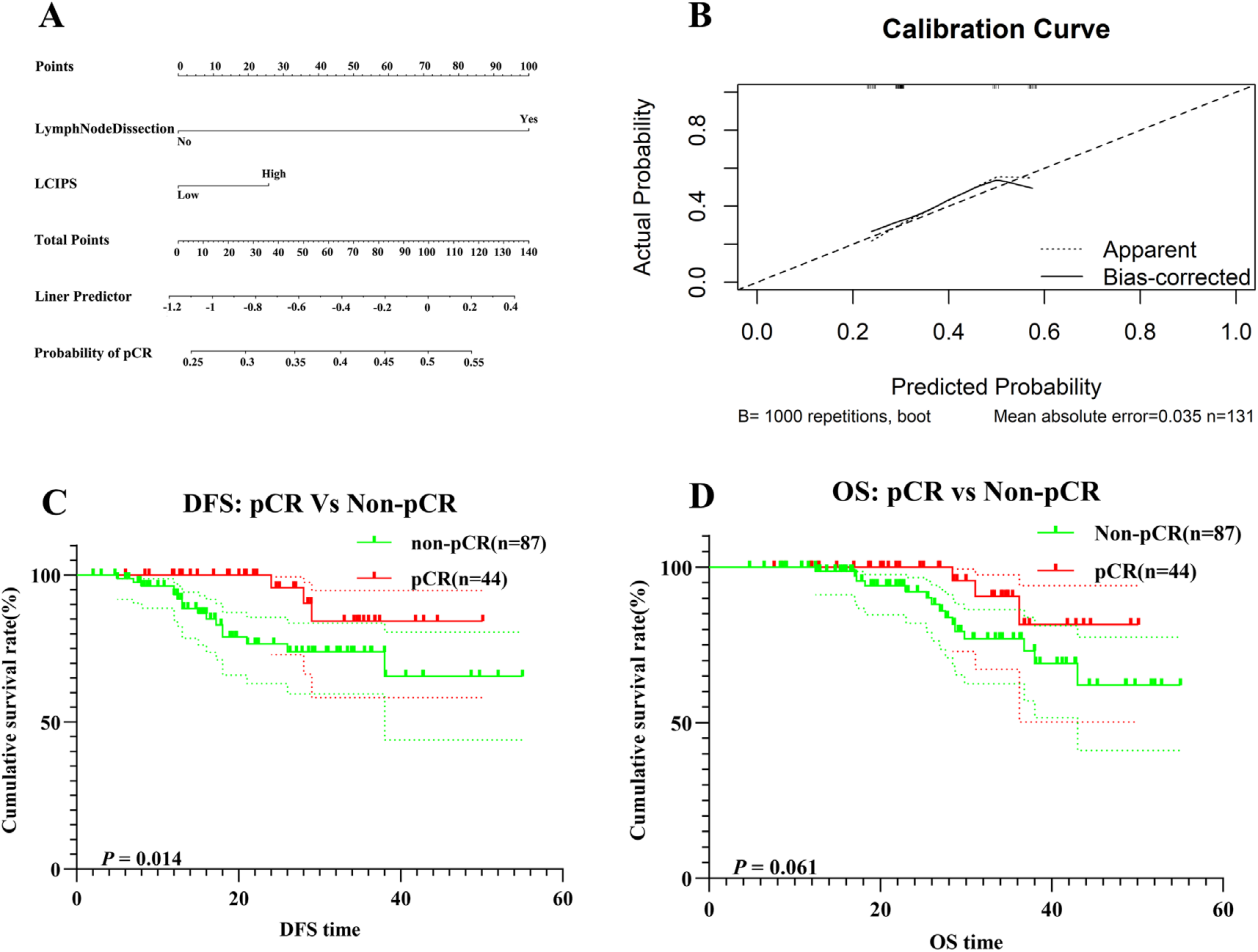
**(A)** The nomogram for pCR prediction; **(B)** Calibration prediction internally for pCR prediction; **(C)** DFS for patients with pCR and non-pCR; **(D)** OS for patients with pCR and non-pCR.

Postoperative pathological examination revealed that 44 (33.6%) patients achieved pCR. Multivariate logistic regression analysis identified the intraoperative lymph node dissection status and LCIPS as independent predictors of pCR (**Table 3**). The absence of lymph node dissection (HR = 3.762, 95% CI: 1.158–12.228, *p* = 0.028), and low LCIPS group (HR = 0.658, 95% CI: 0.407–0.859; *p* = 0.032) were significantly associated with higher pCR rates. A nomogram (**Figure 4A**) constructed using these predictors achieved a C-index value of 0.713 (by bootstrap validation n = 1000). Calibration curves generated through internal bootstrap validation (n = 1000) (**Figure 4B**) demonstrated good model consistency.

**Table 3:** Logistic regression analysis.

Characteristics	Univariate Logistic		Multivariable Logistic	
	*P -*value (FDR-corrected)	HR (95%CI)	*P* -value (FDR-corrected)	HR (95%CI)
Sex (Male vs Female)	0.028 (0.034)	2.571 (1.106-5.979)	0.060 (0.079)	3.142 (0.952-10.367)
Neoadjuvant cycles				
2	Ref		Ref	
3	0.764 (0.764)	1.131 (0.506-2.531)	0.066 (0.079)	4.579 (0.904-8.195)
≥4	0.014 (0.021)	0.786 (0.283-0.985)	0.031 (0.064)	0.173 (0.035-0.851)
Lymph node dissection (No vs. Yes)	0.009 (0.018)	3.098 (1.326-7.241)	0.028 (0.064)	3.762 (1.158-12.228)
Glu (<5.200 vs. ≥5.200)	0.022 (0.033)	2.410 (1.137-5109)	0.419 (0.419)	3.808 (0.241-11.686)
LCIPS (<1.676 vs. ≥1.676)	0.004 (0.024)	0.547 (0.354-0.952)	0.032 (0.064)	0.658 (0.407-0.859)

### 3.5 Impact of pCR on survival prognosis

In this study, a total of 131 NSCLC patients underwent evaluation after receiving nICT. Among them, 87 patients (65.6%) did not achieve a pCR, while only 44 patients (34.4%) attained pCR.

According to the DFS Kaplan-Meier survival analysis (**Figure 4C**), patients in the pCR group exhibited significantly better DFS compared to the non-pCR group (log-rank *p* = 0.014). However, no statistically significant difference in OS (**Figure 4D**) was observed between the pCR and non-pCR groups (log-rank *p* = 0.061).

The 1-year and 3-year OS rates in the pCR group were 98.7% (95% CI: 96.1%–100.0%) and 84.3% (95% CI: 69.3%–93.1%), respectively, whereas in the non-pCR group, the corresponding OS rates were 93.3% (95% CI: 87.7%–99.2%) and 73.8% (95% CI: 62.8%–86.8%), respectively.

### 3.6 Cox regression analysis

Cox regression analysis was conducted for LCIPS and other clinical factors. Results indicated that smoking, SCC, NSE, and LCIPS were significantly associated with DFS (**Table 4**). Multivariate Cox regression analysis revealed that smoking (HR = 18.550, *p* = 0.019), NSE (HR = 0.018, *p* = 0.030), and LCIPS (HR = 13.604, *p* = 0.045) were independent predictors of DFS. Similarly, OS analysis demonstrated that smoking, NEU, SCC, NSE, LDH, and LCIPS were significantly associated with OS (*p* < 0.05). Multivariate analysis identified smoking (HR = 10.752, *p* = 0.005), NEU (HR = 1.574, *p* = 0.049), LDH (HR = 5.624, *p* = 0.034), and LCIPS (HR = 5.721, *p* = 0.042) as independent prognostic factors influencing OS.

**Table 4:** Cox regression analysis.

Characteristics	DFS				OS			
	Univariate Cox		Multivariable Cox		Univariate Cox		Multivariate Cox	
	P -value (FDR-corrected)	HR (95%CI)	P -value (FDR-corrected)	HR (95%CI)	P -value (FDR-corrected)	HR (95CI%)	P -value (FDR-corrected)	HR (95CI%)
Smoking (No vs. Yes)	0.047 (0.071)	2.665 (1.012-7.018)	0.019 (0.090)	18.550 (2.158-20.567)	0.006 (0.032)	2.482 (1.943-6.537)	0.005 (0.030)	10.752 (5.969-24.627)
NEU (<4.550 vs. ≥4.550)	0.968 (0.968)	1.019 (0.414-2.509)	0.080 (0.110)	0.055 (0.002-1.422)	0.032 (0.038)	1.148 (1.461-2.859)	0.049 (0.068)	1.574 (1.237-3.964)
LDH (<177 vs. ≥177)	0.558 (0.670)	0.764 (0.310-1.883)	0.092 (0.110)	0.073 (0.003-1.154)	0.025 (0.038)	1.236 (1.071-2.138)	0.034 (0.068)	5.624 (0.231-10.737)
SCC (<1.400 vs. ≥1.400)	0.033 (0.066)	3.038 (1.091-8.455)	0.406 (0.406)	2.685 (0.261-5.581)	0.045 (0.045)	2.613 (1.941-7.256)	0.863 (0.869)	1.339 (0.049-6.553)
NSE (<15.950 vs. ≥15.950)	0.012 (0.036)	0.456 (0.173-0.702)	0.030 (0.090)	0.018 (0.001-0.673)	0.016 (0.032)	0.388 (0.147-1.025)	0.057 (0.068)	0.012 (0.005-1.936)
LCIPS (<1.676 vs. ≥1.676)	0.001 (0.006)	4.462 (1.788-11.130)	0.045 (0.090)	13.604 (1.024-16.371)	0.013 (0.032)	3.166 (1.280-7.831)	0.042 (0.068)	5.721 (1.552-9.029)

### 3.7 Kaplan-Meier survival analysis

**Figure 5:**
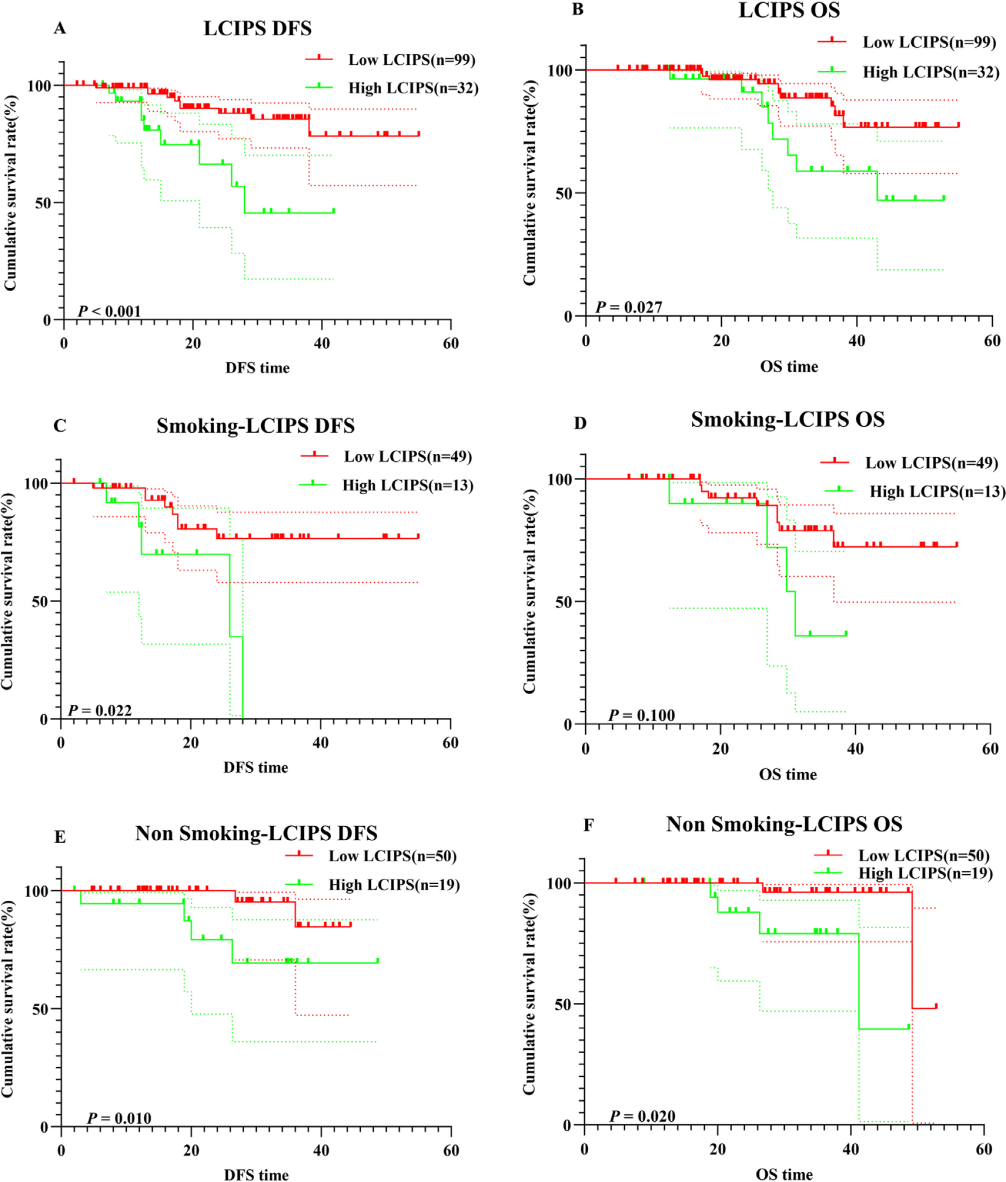
K–M survival curves. **(A)** K–M survival curves of LCIPS for DFS; **(B)** K–M survival curves of LCIPS for OS; **(C)** K–M survival curves of LCIPS for DFS in with Smoking; **(D)** K–M survival curves of LCIPS for OS with Smoking; **(E)** K–M survival curves of LCIPS for DFS in with No Smoking; **(F)** K–M survival curves of LCIPS for OS in with No Smoking.

Kaplan-Meier survival analysis revealed that higher LCIPS values were associated with worse DFS (χ² = 11.51, log-rank *p* < 0.001) and OS (χ² = 4.919, log-rank *p* = 0.027) (**Figure 5**). The 1-year and 3-year DFS rates were 98.9% (95% CI: 96.9%–100.0%) and 85.5% (95% CI: 76.8%–95.2%), respectively, which were significantly higher than those in the high LCIPS group (log-rank *p* = 0.012). The 1-year and 3-year OS rates were 99.2% (95% CI: 97.9%–100.0%) and 85.3% (95% CI: 75.8%–100.0%), respectively. Among 62 patients with a smoking history (low LCIPS group = 49; high LCIPS group = 13), high LCIPS group was associated with shorter DFS (χ² = 5.270, log-rank *p* = 0.022), but it was not significantly related to OS. Conversely, among 69 non-smoking patients (low LCIPS group = 50; high LCIPS group = 19), low LCIPS group was significantly associated with better DFS (χ² = 6.608, log-rank *p* = 0.010) and OS (χ² = 5.384, log-rank *p* = 0.020) (**Figure 5**).

### 3.8 Construction and validation of nomograms

**Figure 6:**
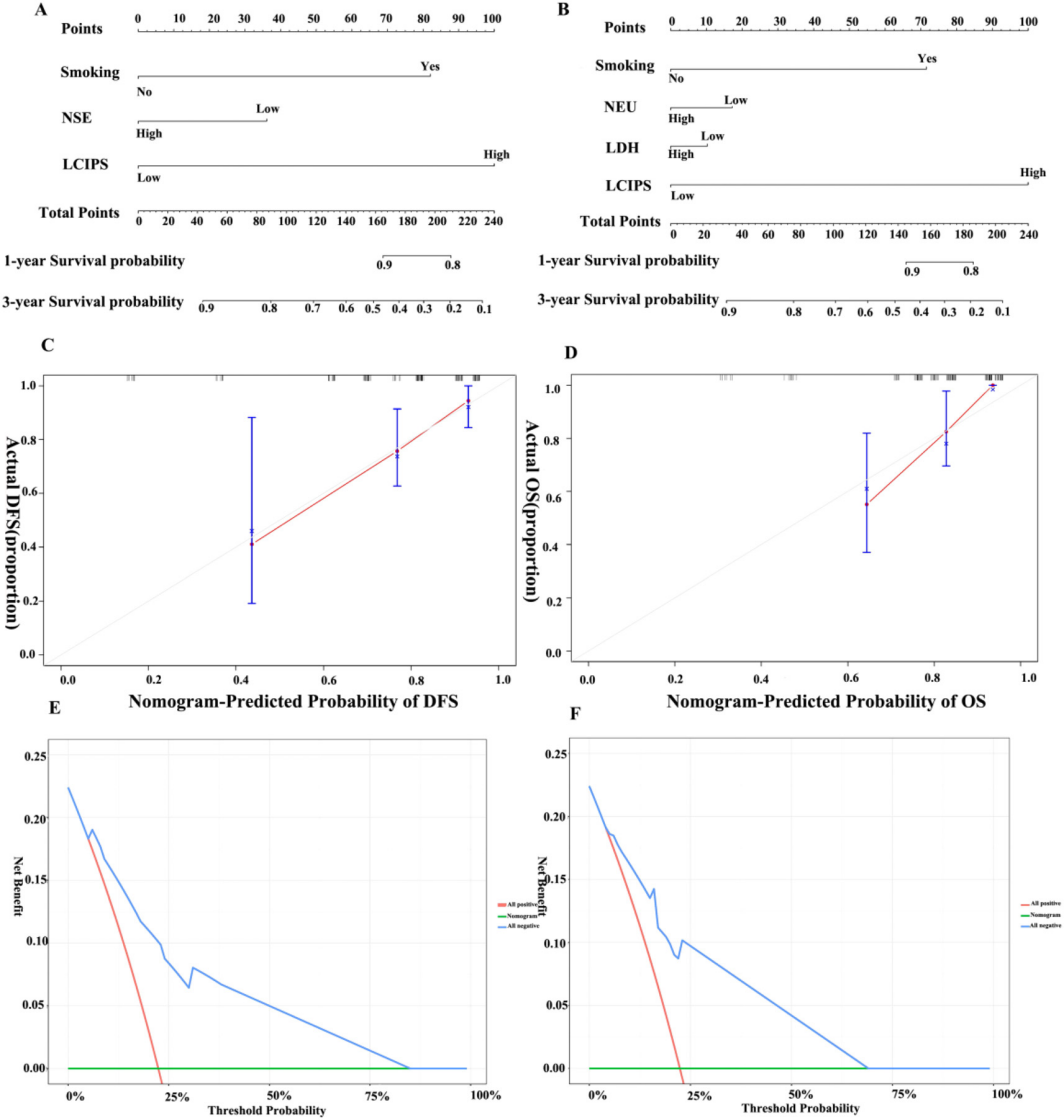
Nomograms for DFS and OS. **(A)** Nomogram for DFS; **(B)** Nomogram for OS. **(C)** Calibration curve for DFS; **(D)** Calibration curve for OS; **(E)** Decision curve for DFS; **(F)** Decision curve for OS.

Nomograms were constructed based on the multivariate analysis results to predict DFS and OS (**Figure 6**), with C-index values of 0.769 and 0.775, respectively. Validation using DCA demonstrated that the nomogram provided a reliable predictive performance and significant clinical applicability.

### 3.9 Safety analysis

**Figure 7:**
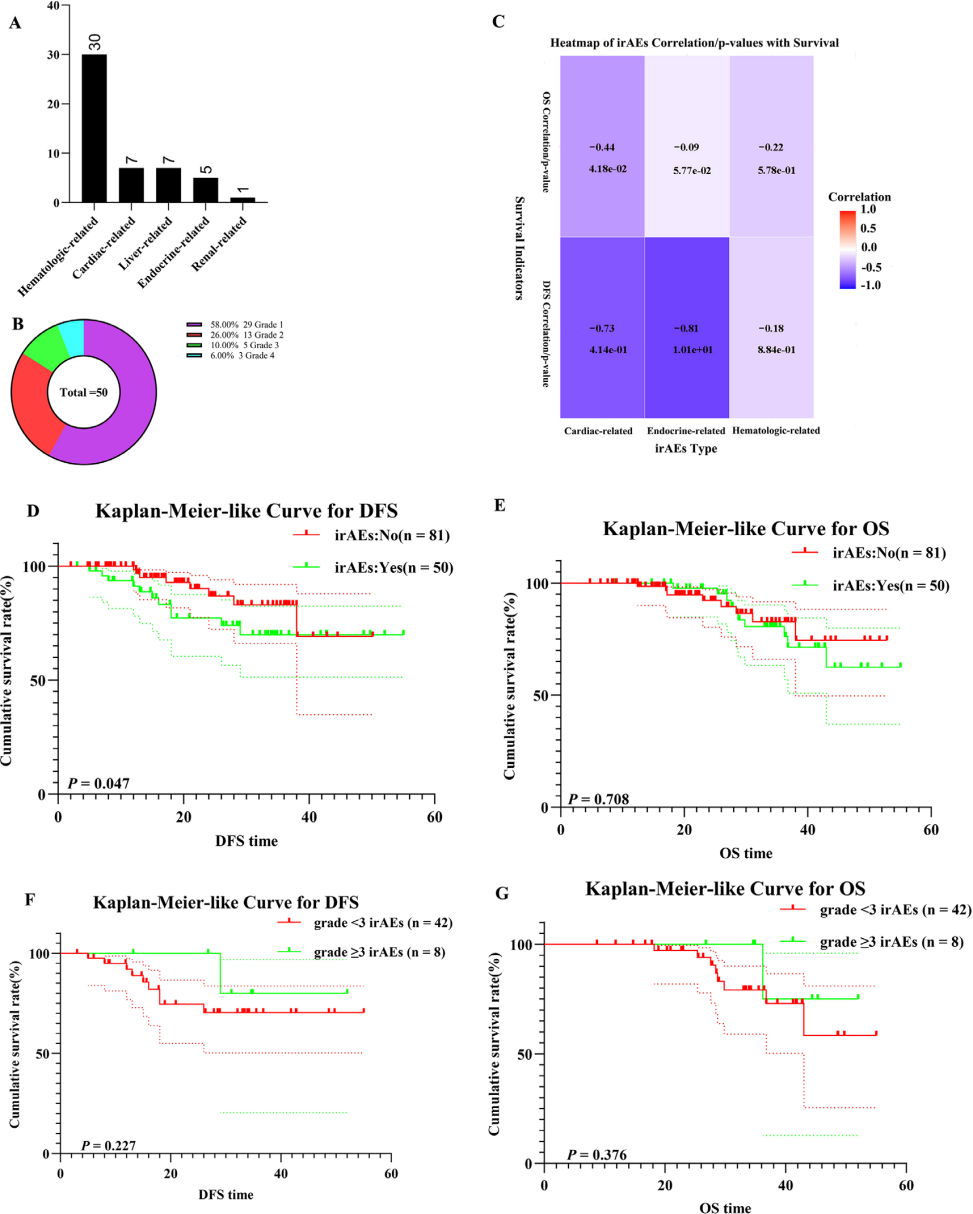
Safety analysis. **(A)** Bar Chart of irAEs; **(B)** Doughnut Chart of irAEs; **(C)** Heatmap of irAEs Correlation with DFS and OS; **(D)** K–M survival curves of irAEs patients for DFS; **(E)** K–M survival curves of irAEs patients for OS; **(F)** K–M survival curves of different grade irAEs patients for DFS; **(G)** K–M survival curves of different grade irAEs patients for OS.

Of the NSCLC patients treated with nICT, 50 experienced irAEs, with hematologic-related irAEs being the most common (n = 30) (**Figure 7**). Eight patients developed grade ≥3 irAEs. To assess the association between specific irAE types and survival outcomes, a correlation heatmap (**Figure 7C**) was generated using Spearman’s rank correlation analysis. Endocrine- and cardiac-related irAEs demonstrated significant negative correlations with DFS (r = -0.81 and r = -0.73), while other irAE subtypes showed no significant associations. Patients with irAEs exhibited shorter DFS than those without irAEs (χ² = 5.120, log-rank *p* = 0.047) (**Figure 7D**). When comparing patients with grade ≥3 irAEs to those with lower-grade irAEs, no significant difference was observed in DFS and OS (χ² = 1.182, log-rank *p* = 0.227; χ² = 0.784,log-rank *p* = 0.376) (**Figures 7G, H**).

## 4 Discussion

The 2024 NCCN guidelines strongly recommend neoadjuvant nivolumab combined with nICT for NSCLC patients with tumors ≥4 cm or positive lymph nodes, provided that there are no contraindications to immune checkpoint inhibitors (33). Clinical trials such as NADIM (34), Neotorch (35), and AEGEAN (12) have demonstrated the safety, feasibility, and efficacy of nICT in resectable stage IB-IIIA NSCLC. These trials have reported promising outcomes, including major pathological response (MPR), pCR, and tumor downstaging, highlighting the potential benefits of nICT. Furthermore, the NEOSTAR (36) study revealed that nICT has minimal adverse effects on surgical resection rates, complexity, and perioperative outcomes. As surrogate endpoints for survival, the MPR and pCR have been validated as key indicators of nICT efficacy (37). Consistent with previous studies, our research showed that patients who achieved pCR exhibited significantly better DFS than those who did not (log-rank *p* = 0.014) (13), underscoring the potential of pCR as a prognostic marker for survival.

This study systematically analyzed the treatment outcomes and survival prognoses of 131 patients with NSCLC who underwent nICT. Using LASSO regression, we developed LCIPS, a novel hematological metric incorporating A/G, MONO, and LYM. The LCIPS model exhibits modest predictive capacity for pCR and DFS, though its performance remains limited, particularly when compared to established conventional biomarkers. Intraoperative lymph node dissection status, A/G, and LCIPS were identified as significant predictors of pCR, while multivariate analyses confirmed LCIPS, smoking history, and tumor markers, such as NSE and LDH, as independent prognostic factors for DFS and OS. Kaplan-Meier survival analysis and nomogram validation further demonstrated the clinical applicability and predictive reliability of LCIPS.

The LCIPS, composed of A/G (38, 39), absolute monocyte count, and absolute lymphocyte count (40), has been demonstrated to predict the survival outcomes of patients with NSCLC. Monocytes can differentiate into macrophages, which play a critical role in the phagocytosis and clearance of cellular debris and pathogens (41). In tumors, tumor-associated macrophages (TAMs) are an essential component of the tumor microenvironment, contributing to tumor progression and drug resistance by creating an immune-suppressive microenvironment (42). For instance, the overexpression of immune checkpoint ligand B7-H1 (PD-L1) on tumor cells is recognized as a key immune evasion mechanism. These PD-L1+ TAMs are activated by tumor-derived IL-10 and mediate CD8+ T cell dysfunction through the PD-1/PD-L1 interaction. Additionally, the blockade of checkpoint molecules on TAMs in ICIs therapies has garnered increasing attention. Gordon et al. demonstrated that PD-1/PD-L1 inhibitors could restore the phagocytic ability of PD-1+ macrophages, thereby prolonging survival in preclinical colorectal cancer models in a macrophage-dependent manner (43).

In immune therapy responses, T lymphocytes, especially CD8+ T cells, are key mediators of therapeutic efficacy. CD8+ T cells directly recognize and kill tumor cells through intracellular antigens, thus inhibiting tumor proliferation and metastasis. As antigen-specific effector cells, the number of CD8+ T cells is considered a marker of cancer regression (44). Kang et al. constructed a T lymphocyte depletion (TLP) mouse model and found that the anti-tumor effect of PD-1 therapy was severely impaired in TLP mice, depending on the degree of TLP and the immunogenicity of the tumor. Moreover, TLP led to alterations in the composition of tumor-infiltrating lymphocytes, with a reduction in PD-1+ tumor-reactive CD8+ T cells (45).

These findings underscore the importance of immune cells, particularly TAMs and CD8+ T cells, in modulating the response to immune therapies. Understanding the interaction between LCIPS components, such as lymphocytes and macrophages, and treatment outcomes is crucial for predicting therapeutic responses and survival in patients receiving immunotherapy.

Among these, absolute lymphocyte count plays a critical role in the tumor microenvironment (TME). Hu et al. utilized single-cell sequencing technology to investigate the TME changes in patients who achieved MPR or non-MPR after nICT (46). Their study revealed that the transcriptional characteristics of FCRL4+FCRL5+ memory B cells and CD16+CX3CR1+ monocytes were enriched in MPR patients, serving as predictive markers of immunotherapy response (46). Similarly, Hui et al. observed that PD-1 inhibitors enhanced the efficacy of neoadjuvant chemotherapy for NSCLC by increasing the number of CD127+ and KLRG1+ CD8 T cells (47). Additionally, this study identified smoking as a critical factor influencing survival outcomes in patients treated with nICT, which is consistent with previous research. Notably, smoking signature were superior to PD-L1 expression in predicting pathological response to neoadjuvant immunotherapy in lung cancer patients (48). Although preoperative A/G has been reported as a prognostic indicator for resectable NSCLC (39, 49), no study has demonstrated its utility as a predictor of pCR. Our findings indicate that a higher A/G ratio predicts pCR status more effectively.

Dynamic monitoring of tumor-related markers provides real-time and precise information for assessing treatment efficacy and predicting prognosis. Previous studies have shown that dynamic changes in peripheral blood inflammatory biomarkers reflect treatment responses in patients with NSCLC undergoing nICT and are closely associated with prognosis. For instance, elevated post-treatment levels of NLR, PLR, SII, and modified Glasgow Prognostic Score (mGPS), and increases in PLR and δSII were significantly correlated with worse OS and EFS (20). Furthermore, the dynamic monitoring of circulating tumor DNA (ctDNA) offers additional insights into the efficacy of nICT. A multicenter, open-label, phase II study (CTONG1804) demonstrated that preoperative neoadjuvant therapy and postoperative ctDNA-negative status might predict better pathological responses and survival benefits (11). With advancements in single-cell sequencing technology, increasing evidence has revealed dynamic TME changes following nICT. Using single-cell sequencing, Yang et al. found that the post-treatment TME of non-responders with esophageal squamous cell carcinoma was enriched with CXCL13+CD8+Tex cells (exhausted phenotype) and TNFRSF4+CD4+ Tregs (immunosuppressive function) (50). Importantly, LCIPS proposed in this study potentially complements these dynamic monitoring methods. As treatment progresses, dynamic changes in A/G, monocytes, and lymphocytes may further enhance LCIPS’s predictive performance for DFS and OS. Therefore, integrating LCIPS with dynamic monitoring methods could further optimize the accuracy of treatment outcome predictions.

Although this study provides important evidence for the construction and application of LCIPS, it has certain limitations. First, as this was a single-center retrospective study, potential information bias exists, and multicenter prospective studies are required to validate our findings. Second, the small sample size may limit the generalizability of our conclusions, which necessitates further research with larger cohorts. Third, the short follow-up period restricted the assessment of the long-term survival outcomes. Fourth, the LCIPS cut-off value was derived using ROC analysis, which may have been influenced by sample characteristics. Future studies should explore dynamic assessment approaches to enhance sensitivity and stability. Fifth, missing data may have introduced selection bias, although we employed multiple imputation methods to mitigate this issue. However, residual confounding cannot be entirely ruled out. Future research should focus on improving data completeness and assessing the impact of missing data on predictive performance. Finally, the LCIPS was constructed and validated using internal datasets, lacking external independent validation, which future multicenter studies should address.

## 5 Conclusions

The LCIPS is a novel predictive index for preoperative nICT that effectively predicts pCR and survival outcomes in patients with NSCLC. This index offers a valuable foundation for the development of personalized treatment strategies and demonstrates robust clinical applicability and predictive reliability.

## Data Availability

The original contributions presented in the study are included in the article/**Supplementary Material**. Further inquiries can be directed to the corresponding author.

## References

[1] Siegel RL Giaquinto AN Jemal A. Cancer statistics, 2024. CA Cancer J Clin. (2024) 74:12–49. doi: 10.3322/caac.21820 38230766

[2] Chen P Liu Y Wen Y Zhou C. Non-small cell lung cancer in China. Cancer Commun (Lond). (2022) 42:937–70. doi: 10.1002/cac2.v42.10 PMC9558689 36075878

[3] Rajaram R Huang Q Li RZ Chandran U Zhang Y Amos TB. Recurrence-free survival in patients with surgically resected non-small cell lung cancer: A systematic literature review and meta-analysis. Chest. (2024) 165:1260–70. doi: 10.1016/j.chest.2023.11.042 38065405

[4] Potter AL Costantino CL Suliman RA Haridas CS Senthil P Kumar A. Recurrence after complete resection for non-small cell lung cancer in the national lung screening trial. Ann Thorac Surg. (2023) 116:684–92. doi: 10.1016/j.athoracsur.2023.06.004 37356517

[5] Preoperative chemotherapy for non-small-cell lung cancer: a systematic review and meta-analysis of individual participant data. Lancet. (2014) 383(9):1561–71. doi: 10.1016/S0140-6736(13)62159-5 PMC4022989 24576776

[6] Kang J Zhang C Zhong WZ. Neoadjuvant immunotherapy for non-small cell lung cancer: State of the art. Cancer Commun (Lond). (2021) 41:287–302. doi: 10.1002/cac2.12153 33689225 PMC8045926

[7] Forde PM Spicer J Lu S Provencio M Mitsudomi T Awad MM. Neoadjuvant nivolumab plus chemotherapy in resectable lung cancer. N Engl J Med. (2022) 386:1973–85. doi: 10.1056/NEJMoa2202170 PMC9844511 35403841

[8] Sorin M Prosty C Ghaleb L Nie K Katergi K Shahzad MH. Neoadjuvant chemoimmunotherapy for NSCLC: A systematic review and meta-analysis. JAMA Oncol. (2024) 10:621–33. doi: 10.1001/jamaoncol.2024.0057 PMC10958389 38512301

[9] Deng H Zhao Y Cai X Chen H Cheng B Zhong R. PD-L1 expression and Tumor mutation burden as Pathological response biomarkers of Neoadjuvant immunotherapy for Early-stage Non-small cell lung cancer: A systematic review and meta-analysis. Crit Rev Oncol Hematol. (2022) 170:103582. doi: 10.1016/j.critrevonc.2022.103582 35031441

[10] Forde PM Chaft JE Smith KN Anagnostou V Cottrell TR Hellmann MD. Neoadjuvant PD-1 blockade in resectable lung cancer. N Engl J Med. (2018) 378:1976–86. doi: 10.1056/NEJMoa1716078 PMC6223617 29658848

[11] Liu SY Dong S Yang XN Liao RQ Jiang BY Wang Q. Neoadjuvant nivolumab with or without platinum-doublet chemotherapy based on PD-L1 expression in resectable NSCLC (CTONG1804): a multicenter open-label phase II study. Signal Transduct Target Ther. (2023) 8:442. doi: 10.1038/s41392-023-01700-4 38057314 PMC10700550

[12] Heymach JV Harpole D Mitsudomi T Taube JM Galffy G Hochmair M. Perioperative durvalumab for resectable non-small-cell lung cancer. N Engl J Med. (2023) 389:1672–84. doi: 10.1056/NEJMoa2304875 37870974

[13] Zhai WY Duan FF Lin YB Lin YB Zhao ZR Wang JY. Pan-immune-inflammatory value in patients with non-small-cell lung cancer undergoing neoadjuvant immunochemotherapy. J Inflammation Res. (2023) 16:3329–39. doi: 10.2147/JIR.S418276 PMC10422963 37576157

[14] Tao X Zhang Q Yuan P Wang S Ying J Li N. Predictive value of longitudinal systemic inflammatory markers for pathologic response to neoadjuvant PD-1 blockade in resectable non-small cell lung cancer. Transl Lung Cancer Res. (2024) 13:2972–86. doi: 10.21037/tlcr-24-598 PMC11632438 39670003

[15] Feng J Wang L Yang X Chen Q Cheng X. Comprehensive nutritional index predicts clinical outcomes for esophageal squamous cell carcinoma receiving neoadjuvant immunotherapy combined with chemotherapy. Int Immunopharmacol. (2023) 121. doi: 10.1016/j.intimp.2023.110459 37307758

[16] Feng J Wang L Yang X Chen Q Cheng X. Pathologic complete response prediction to neoadjuvant immunotherapy combined with chemotherapy in resectable locally advanced esophageal squamous cell carcinoma: real-world evidence from integrative inflammatory and nutritional scores. J Inflammation Res. (2022) 15:3783–96. doi: 10.2147/JIR.S367964 PMC9271687 35832830

[17] Basher F Saravia D Lopes G. Prognostic value of systemic inflammatory markers in first- and subsequent-line immunotherapy and durability of response in NSCLC. J Clin Oncol. 39:e21210–0. doi: 10.1200/jco.2021.39.15_suppl.e21210

[18] Zhou K Cao J Lin H Liang L Shen Z Wang L. Prognostic role of the platelet to lymphocyte ratio (PLR) in the clinical outcomes of patients with advanced lung cancer receiving immunotherapy: A systematic review and meta-analysis. Front Oncol. (2022) 12:962173. doi: 10.3389/fonc.2022.962173 36059629 PMC9437586

[19] Shi Y Liu X Liu J Zhang D Liu X Yue Y. Correlations between peripheral blood biomarkers and clinical outcomes in advanced non-small cell lung cancer patients who received immunotherapy-based treatments. Transl Lung Cancer Res. (2021) 10:4477–93. doi: 10.21037/tlcr-21-710 PMC8743518 35070755

[20] Huai Q Luo C Song P Bie F Bai G Li Y. Peripheral blood inflammatory biomarkers dynamics reflect treatment response and predict prognosis in non-small cell lung cancer patients with neoadjuvant immunotherapy. Cancer Sci. (2023) 114:4484–98. doi: 10.1111/cas.v114.12 PMC10728017 37731264

[21] Travis WD Dacic S Wistuba I Sholl L Adusumilli P Bubendorf L. IASLC multidisciplinary recommendations for pathologic assessment of lung cancer resection specimens after neoadjuvant therapy. J Thorac Oncol. (2020) 15:709–40. doi: 10.1016/j.jtho.2020.01.005 PMC8173999 32004713

[22] Mountzios G Remon J Hendriks LEL García-Campelo R Rolfo C Van Schil P. Immune-checkpoint inhibition for resectable non-small-cell lung cancer - opportunities and challenges. Nat Rev Clin Oncol. (2023) 20:664–77. doi: 10.1038/s41571-023-00794-7 37488229

[23] Jerome Friedman THA Tibshirani R Balasubramanian N Tay K Simon N Qian J. glmnet: lasso and elastic-net regularized generalized linear models. (2023).

[24] rms: regression modeling strategies (2025).

[25] Xavier Robin A Turck N Hainard A Tiberti N Lisacek F Sanchez J-C. Zane Billings(DeLong paired test CI). In: pROC: display and analyze ROC curves (2023). Fast DeLong code.

[26] Daniel C Sjoberg D Vertosick E. dcurves: decision curve analysis for model evaluation. (2024).

[27] Kolde R. pheatmap: pretty heatmaps. (2019).

[28] Salciccia S Frisenda M Bevilacqua G Viscuso P Casale P De Berardinis E. Prognostic value of albumin to globulin ratio in non-metastatic and metastatic prostate cancer patients: A meta-analysis and systematic review. Int J Mol Sci 23. (2022) 23(19):11501. doi: 10.3390/ijms231911501 PMC9570150 36232828

[29] Cassetta L Fragkogianni S Sims AH Swierczak A Forrester LM Zhang H. Tumor-associated macrophage and monocyte transcriptional landscapes reveal cancer-specific reprogramming Biomarkers, and therapeutic targets. Cancer Cell. (2019) 35:588–602.e10. doi: 10.1016/j.ccell.2019.02.009 30930117 PMC6472943

[30] Arwert EN Harney AS Entenberg D Wang Y Sahai E Pollard JW. A unidirectional transition from migratory to perivascular macrophage is required for tumor cell intravasation. Cell Rep. (2018) 23:1239–48. doi: 10.1016/j.celrep.2018.04.007 PMC5946803 29719241

[31] Jung K Heishi T Khan OF Kowalski PS Incio J Rahbari NN. Ly6Clo monocytes drive immunosuppression and confer resistance to anti-VEGFR2 cancer therapy. J Clin Invest. (2017) 127:3039–51. doi: 10.1172/JCI93182 PMC5531423 28691930

[32] Ménétrier-Caux C Ray-Coquard I Blay JY Caux C. Lymphopenia in Cancer Patients and its Effects on Response to Immunotherapy: an opportunity for combination with Cytokines? J Immunother Cancer. (2019) 7:85. doi: 10.1186/s40425-019-0549-5 30922400 PMC6437964

[33] N.C.C. Network. Non-small cell lung cancer - early and locally advanced. National Comprehensive Cancer Network (2024).

[34] Provencio M Nadal E Insa A García-Campelo MR Casal-Rubio J Dómine M. Neoadjuvant chemotherapy and nivolumab in resectable non-small-cell lung cancer (NADIM): an open-label, multicentre, single-arm, phase 2 trial. Lancet Oncol. (2020) 21:1413–22. doi: 10.1016/S1470-2045(20)30453-8 32979984

[35] Lu S Zhang W Wu L Wang W Zhang P Fang W. Perioperative toripalimab plus chemotherapy for patients with resectable non-small cell lung cancer: the neotorch randomized clinical trial. Jama. (2024) 331:201–11. doi: 10.1001/jama.2023.24735 PMC10792477 38227033

[36] Cascone T William WN Jr. Weissferdt A Leung CH Lin HY Pataer A. Neoadjuvant nivolumab or nivolumab plus ipilimumab in operable non-small cell lung cancer: the phase 2 randomized NEOSTAR trial. Nat Med. (2021) 27:504–14. doi: 10.1038/s41591-020-01224-2 PMC8818318 33603241

[37] Hines JB Cameron RB Esposito A Kim L Porcu L Nuccio A. Evaluation of major pathologic response and pathologic complete response as surrogate end points for survival in randomized controlled trials of neoadjuvant immune checkpoint blockade in resectable in NSCLC. J Thorac Oncol. (2024) 19:1108–16. doi: 10.1016/j.jtho.2024.03.010 PMC12697759 38461929

[38] Lu P Ma Y Wei S Liang X. A low albumin-to-globulin ratio predicts a poor prognosis in patients with metastatic non-small-cell lung cancer. Front Med (Lausanne). (2021) 8:621592. doi: 10.3389/fmed.2021.621592 33732716 PMC7956965

[39] Wu W Zhang L Wang C Xu Z Feng C Zhang Z. The prognostic value of the preoperative albumin/globulin and monocyte ratio in resected early-stage non-small cell lung cancer. Asian J Surg. (2024) 47:118–23. doi: 10.1016/j.asjsur.2023.06.068 37419798

[40] Su S Chen F Lv X Qi L Ding Z Ren W. Predictive value of peripheral blood biomarkers in patients with non-small-cell lung cancer responding to anti-PD-1-based treatment. Cancer Immunol Immunother. (2024) 73:12. doi: 10.1007/s00262-023-03620-2 38231411 PMC10794255

[41] Goswami S Anandhan S Raychaudhuri D Sharma P. Myeloid cell-targeted therapies for solid tumours. Nat Rev Immunol. (2023) 23:106–20. doi: 10.1038/s41577-022-00737-w 35697799

[42] Li M Yang Y Xiong L Jiang P Wang J Li C. Metabolism, metabolites, and macrophages in cancer. J Hematol Oncol. (2023) 16:80. doi: 10.1186/s13045-023-01478-6 37491279 PMC10367370

[43] Xiang X Wang J Lu D Xu X. Targeting tumor-associated macrophages to synergize tumor immunotherapy. Signal Transduct Target Ther. (2021) 6:75. doi: 10.1038/s41392-021-00484-9 33619259 PMC7900181

[44] Xie Q Ding J Chen Y. Role of CD8(+) T lymphocyte cells: Interplay with stromal cells in tumor microenvironment. Acta Pharm Sin B. (2021) 11:1365–78. doi: 10.1016/j.apsb.2021.03.027 PMC8245853 34221857

[45] Kang YW Choi D Moon D Lee KJ Oh Y Yang J. Enabling immune checkpoint blockade efficacy in T-lymphopenia by restoring CD8 T cell dynamics with IL-7 cytokine therapy. Front Immunol. (2024) 15:1477171. doi: 10.3389/fimmu.2024.1477171 39763661 PMC11701376

[46] Hu J Zhang L Xia H Yan Y Zhu X Sun F. Tumor microenvironment remodeling after neoadjuvant immunotherapy in non-small cell lung cancer revealed by single-cell RNA sequencing. Genome Med. (2023) 15:14. doi: 10.1186/s13073-023-01164-9 36869384 PMC9985263

[47] Hui Z Ren Y Zhang D Chen Y Yu W Cao J. PD-1 blockade potentiates neoadjuvant chemotherapy in NSCLC via increasing CD127(+) and KLRG1(+) CD8 T cells. NPJ Precis Oncol. (2023) 7:48. doi: 10.1038/s41698-023-00384-x 37231145 PMC10213055

[48] Yang H Ma W Sun B Fan L Xu K Hall SRR. Smoking signature is superior to programmed death-ligand 1 expression in predicting pathological response to neoadjuvant immunotherapy in lung cancer patients. Transl Lung Cancer Res. (2021) 10:3807–22. doi: 10.21037/tlcr-21-734 PMC8512473 34733630

[49] Zhang H Zhang B Zhu K Wu C Gao L Sun X. Preoperative albumin-to-globulin ratio predicts survival in patients with non-small-cell lung cancer after surgery. J Cell Physiol. (2019) 234:2471–9. doi: 10.1002/jcp.v234.3 30317549

[50] Yang Z Tian H Chen X Li B Bai G Cai Q. Single-cell sequencing reveals immune features of treatment response to neoadjuvant immunochemotherapy in esophageal squamous cell carcinoma. Nat Commun. (2024) 15:9097. doi: 10.1038/s41467-024-52977-0 39438438 PMC11496748

